# Turnover intentions among rural-oriented medical students in Western China: a constructivist grounded theory study

**DOI:** 10.1017/S1463423625000039

**Published:** 2025-03-12

**Authors:** Pinghua Zhu, Xinyi Xu, Chun Zhai, Chen He, Yulu Su, Haitao Yuan, Zukang Gong, Zhong Tang

**Affiliations:** 1 College of Humanities and Social Sciences, Guangxi Medical University, Guangxi, China; 2 Nanning Municipal Health Commission, Guangxi, China; 3 Guangxi Medical Management Service Guidance Center, Guangxi, China

**Keywords:** Grounded theory, rural-oriented medical students, turnover

## Abstract

**Objective:**

The rural-oriented tuition-waived medical education program in China, started in 2010, provides free medical education to students committed to serving in rural areas to address medical staff shortages. Despite its success in training and deploying graduates, retaining them post-obligation remains challenging. This study explores the mechanisms behind the turnover intentions of rural-oriented medical students in Western China, offering insights for their retention.

**Methods:**

Semi-structured interviews were conducted with 47 rural-oriented medical students and 30 health clinic directors in Nanning City. Interview data were analysed using grounded theory, and open, axial and selective coding was applied.

**Results:**

Through three levels of coding analysis, 34 tree nodes, 13 sub-categories and 3 main categories were identified from the interviews with rural-oriented medical students and health clinic directors. 3 main categories were Subjective Norms, Behavioural Attitudes, and Perceived Behavioural Control.

**Conclusion:**

A model of turnover intention among rural-oriented medical students was developed. This model can serve as a valuable reference for future policy optimization concerning China’s rural order-directed medical students.

## Strengths and limitations of this study

- The model is constructed based on Grounded Theory rather than through questionnaire-based research, offering a more comprehensive exploration of the mechanisms behind the formation of turnover intentions among rural-oriented medical students.
- This model incorporates the elements of Subjective Norms and Perceived Behavioral Control. These components encompass the unique circumstances encountered in the actual implementation of the RTME program in China.
- The model didn’t involve incorporating demographic and sociological factors.

The shortage of primary healthcare manpower is a pervasive global issue, which is particularly pronounced in rural regions. This shortfall not only constricts the operational capacity of primary healthcare facilities but also impinges on the quality of services delivered. Nations worldwide have adopted a variety of strategies and interventions to respond to this challenge. The World Health Organization has summarized principal intervention measures to include educational, compulsory, economic incentive-based, and management and support categories (Organization, 2010). Previous research has indicated that a well-structured and organized rural medical student program can enhance medical students’ intention to work in rural areas (Amalba *et al.*, 2018; Johnson *et al.*, 2018). Several countries have been implementing free medical student programs targeted at rural areas since the last century. In the United States, state government-backed initiatives such as the Physician Shortage Area Program (PSAP), Rural Education and Action Development (READ) and the Rural Physician Associate Program (RPAP) have been established. They depend on universities to cultivate a generation of rural doctors (Halaas, 2005; Smucny *et al.*, 2005; Wiwanitkit, 2011). Similarly, Thailand has put forth programs like One District One Doctor (ODOD) and the Collective Program to Increase Production of Rural Doctors (CPIRD) to address the same concerns (Putthasri *et al.*, 2013).

In 2010, China began to implement the rural-oriented tuition-waived medical education program (RTME), colleges recruit rural students who sign an agreement with local health administrative departments before admission. After receiving five years of complimentary medical education at the medical college, these rural students fulfill the contract by serving in the healthcare institutions of their places of origin for six years. From 2010–2020, the Chinese government trained over 70,000 rural-oriented free medical students for township health clinics in the central and western regions of China, with an average of 1.9 undergraduate directed medical students per clinic. In 2021, the first batch of students completing their service period had the option to either continue working in their current clinics or leave. Those who chose to stay could help alleviate the shortage of manpower in township health clinics.

Previous studies have explored the implementation outcomes of the rural-oriented medical student program in China. Huang *et al*. found that the evaluation of learning outcomes in the free medical student program was relatively low, with rural-oriented medical students primarily lacking clinical reasoning and hands-on skills (Huang *et al.*, 2019). Shen *et al*. discovered that rural-oriented medical students experience a higher level of academic burnout, Some studies have also focused on the compliance of rural-oriented medical students with service contracts (Shen *et al.*, 2016). Zhang *et al*. found that the fulfillment rate of service contracts among the first cohort of rural-oriented medical students in China was 99.3%, An additional monthly salary of 1,000 yuan in township health clinics can result in rural-oriented medical students staying for an extra year (Zhang *et al.*, 2017), Pei *et al*. conducted a survey on the willingness of rural-oriented students in Gansu Province to work in rural areas, the research found the attitude of rural-oriented medical students towards working in rural areas is mainly influenced by socioeconomic and cultural conditions (Pei *et al.*, 2018), Scholars have also conducted surveys on the professional values of rural-oriented medical students. Zheng *et al*. found that rural-oriented medical students differ from regular medical students in their pursuit of social status and contributions to social advancement (Zheng *et al.*, 2017), Huai et al. discovered a correlation between the sense of life’s meaning and professional identity among free medical students; components of life’s meaning such as life purpose, control over life, and acceptance of suffering influence professional identity (Huai *et al.*, 2020). The job satisfaction of rural-oriented medical students in China is also a hot topic. Yan et al. conducted a survey on rural-oriented medical students in Jiangsu province, China ((Yan *et al.*, 2022), dividing the participants into two groups for analysis: Group A (rural-oriented medical students) and Group B (their colleagues). Group A demonstrated moderate satisfaction. The factors influencing job satisfaction among rural-oriented medical students were identified as monthly income, weekly working hours, professional title, and post. However, there are few studies that systematically and comprehensively explore the formation mechanism of resignation intention of Chinese rural-oriented medical students after the service period expires and summarize the interaction between various influencing factors.

Grounded theory, which incorporates the strengths of quantitative studies into qualitative ones, has increasingly been applied in health policy research and enhances the ‘scientific nature’ of these studies (Corbin and Strauss, 1990; Glaser *et al.*, 1968). In this study, grounded theory was utilized to summarize and analyse the model of the factors influencing the intention to leave among directed medical students after the service period and the relationships in their emergence and development. The aim was to comprehensively summarize the mechanisms behind the generation and formation of turnover intentions among rural-oriented medical students, encouraging more of these students to work at the grassroots level.

## Data sources and methodology

### Participants and setting

As the capital of Guangxi Zhuang Autonomous Region, Nanning City is in the western part of China. With the largest population in the province and a significant demand for primary healthcare services, the city comprises seven districts and five counties. Data analysis showed that County A and City B (a county-level city) experienced the most severe loss of primary healthcare personnel. Moreover, rural-oriented medical student service program has been implemented in these areas for an extended period. The analysis of the factors influencing the resignation of rural-oriented medical students in these two areas is highly representative. In May and June of 2022, all rural-oriented medical students and respective health clinic directors in County A and City B of Nanning City were selected as interviewees. Interviews were conducted until no new themes emerged, which indicated data saturation. In total, 47 directed medical students (26 from County A and 21 from City B) and 30 township health clinic directors (15 from County A and 15 from City B) were interviewed.

### Methods

#### Grounded theory

Grounded theory, initially developed by Glaser and Strauss, is a qualitative research method that incorporates quantitative research elements. It builds theory from data, is suited for addressing complex social phenomena, and explains changes, processes and interactions. Currently, grounded theory has three schools, namely classic procedural and constructivist versions by Glaser and Strauss, Strauss and Corbin, as well as Charmaz, respectively. The procedural version by Strauss and Corbin, often referred to as “classic grounded theory”, employs a three-tier coding process: open, axial and selective coding. Compared to the other versions, the procedural and neutral nature of the approach by Strauss and Corbin has contributed to its widespread use in the field of health policy research (Li *et al.*, 2022; Xu *et al.*, 2022; Zhang *et al.*, 2019). Compared to the other versions, the procedural and neutral nature of the approach by Strauss and Corbin has contributed to its widespread use in the field of health policy research. This study employs the Grounded Theory to explore the mechanisms behind the turnover intentions of rural-oriented medical students. Meanwhile, the interactions and potential outcomes of various factors will also be explained in this paper.

#### Data collection

In this study, semi-structured interviews were initially conducted using an interview outline validated by experts and lasted between 40 and 60 minutes. In addition, they covered topics such as the reasons for enrolling in the rural-oriented free medical education program, work compensation, work environment, career prospects, post-service considerations for leaving, and suggestions for the directed medical student training policy.

### Data analysis

**Figure 1.**
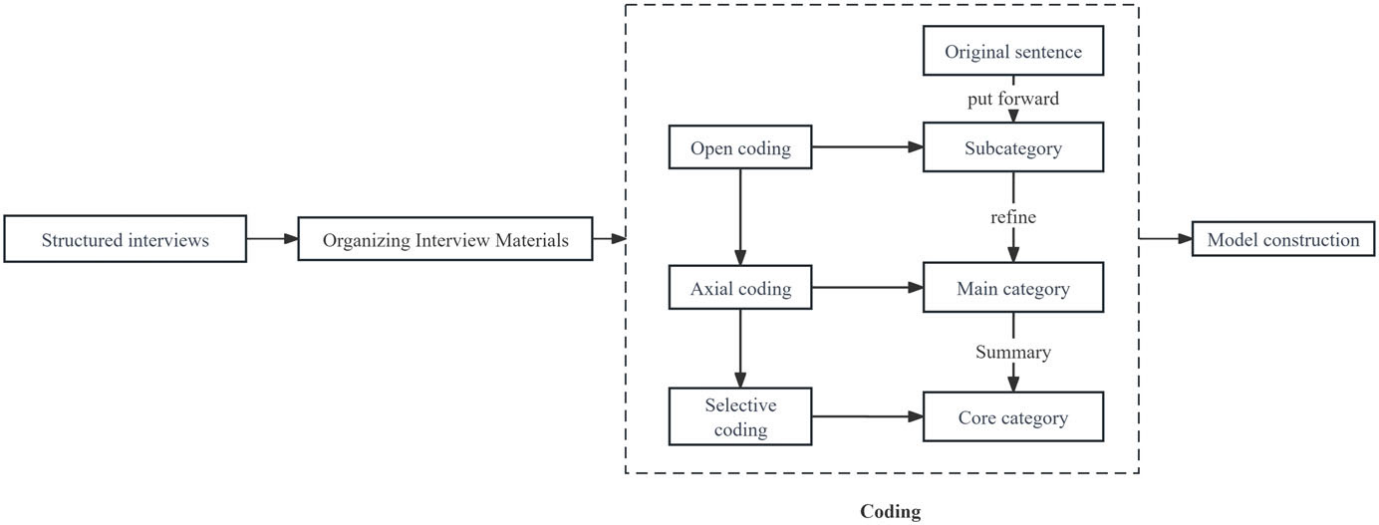
Data collection and analysis.

The collected data were imported into QSR NVivo 12 for coding by two graduate students. Following the procedural grounded theory of Strauss and Corbin, data underwent open, axial and selective coding to identify relevant concepts and categories. This process analyzed the factors influencing the intention to leave among directed medical students in Nanning City after the service period and summarized the relationships in the emergence and development of these factors. This led to the construction of a model framework of the factors influencing the intention to leave. Throughout the analysis, a third party supervised the process until the coding consistency rate of the two graduate students reached over 95%, which formed the final coding result. The data collection and analysis procedures are illustrated in Figure 1.

#### Open coding results

Open coding is the process of categorizing similar concepts from interview data into the same categories. The collected 77 pieces of data were coded by two researchers independently, which resulted in 519 and 487 free nodes respectively, with 479 identical free nodes between them. Based on the formula R (reliability) = M (the number of consistent codes between two researchers)/N (the average number of codes by two researchers), coding reliability was calculated to be 0.95, which indicated good reliability.

#### Axial coding results

Axial coding involves analyzing the logic between categories identified in the initial stage and summarizing the internal relationships between these categories.

#### Selective coding results

Selective coding refers to the process of placing research findings within a broader conceptual framework, verifying these relationships and refining conceptualization. The theory of planned behavior (TPB), a socio-psychological model, can explain changes in human behavioral patterns. TPB examines how these beliefs determine Behavioral attitudes, subjective norms and perceived behavioral control, which in turn influences their behavioral intentions. This article employs selective coding based on TPB to investigate the turnover intentions of rural-oriented medical students, exploring the interactive mechanisms among these elements.

## Results

### Basic information of rural-oriented medical students

Among the 47 rural-oriented medical students interviewed, 17 were male and 30 were female. The longest work experience was six years, and the shortest was one year. A total of 16 directed medical students had an undergraduate degree, including 12 from a provincial medical university and four from a traditional Chinese medicine university. Additionally, 31 students with an associate degree were included: three from a provincial traditional Chinese medicine vocational college and 28 from a provincial health vocational and technical college. Moreover, 31 rural-oriented medical students who accounted for 65.96% of the total expressed an intention to leave after completing their service period. Among them, nine undergraduate students (56.25% of the undergraduate group) and 12 students with an associate degree (38.71% of the associate degree group) indicated an intention to leave. A total of 47 rural-oriented medical students were allocated to 39 township health clinics, with 22 in County A and 17 in City B. Correspondingly, 30 directors from these clinics participated in the interviews.

### Coding results

**Figure 2.**
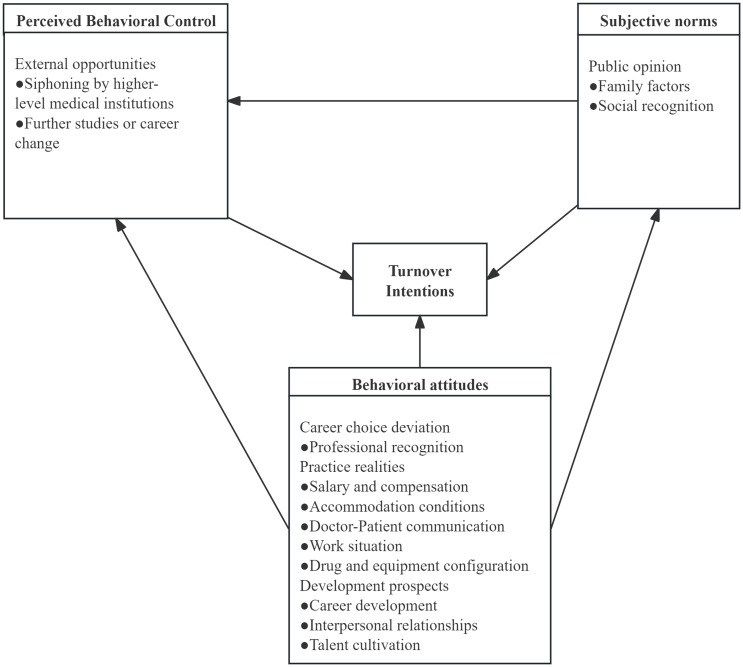
Diagram illustrating the emergence and development of turnover intentions among rural-oriented medical students.

After further analysis and discussion, 483 free nodes were finalized. Then, similar free nodes were refined and summarized, which resulted in 34 tree nodes and 13 sub-categories. They included salary and compensation, interpersonal relationships, accommodation conditions, career development, doctor-patient communication, talent cultivation, professional recognition, work situation, drug and equipment configuration, family factors, social recognition, recruitment by upper-level medical institutions, staying in the health clinic, and pursuing further studies or career change. By synthesis and organization, the 13 sub-categories identified during the open coding phase of the interview data of rural-oriented medical students were summarized into 3 main categories. These categories included public opinion, career choice deviation, professional realities, development prospects and external opportunities. Based on axial coding, the 3 main categories and 13 sub-categories were examined, which resulted in the identification of core categories encompassing all others, presented in Table 1. The core categories identified were subjective norms, behavioral attitudes and perceived behavioral control. A relationship diagram integrating various concepts and categories was developed, as detailed in Figure 2.

**Table 1.** Coding process of turnover intention

		Open coding	
Selective coding	Axial coding	Category	Concept
Subjective norms	Public opinion	Family factors	Difficulty in transferring to hometown clinics Far from home Family pressure to work back home
		Social recognition	Negative public opinions about directed medical students online The spread of negative emotions
Behavioral attitudes	Career choice deviation	Professional recognition	Insufficient understanding of policies, mismatch between parents’ choice and personal expectations Service period longer than expected Inability to pursue a master’s degree
	Professional realities	Salary and compensation	Salary disproportionate to effort The delayed disbursement of general practitioner subsidies Poor welfare benefits Urgent need for adjustment in the salary distribution system Poor autonomy in health clinic management Limited grassroots finance
		Accommodation conditions	Outdated dormitory infrastructure Inconvenience due to shared rooms Distance from workplace and remote locations with few entertainments’ facilities
		Doctor-patient communication	Non-cooperation from patients Difficulties in doctor-patient communication
		Work situation	High workload Low technical level Mismatch between work content and specialization Focus on public health over clinical work.
		Drug and equipment configuration	Outdated medical equipment Insufficient drug supply and old infrastructure
	Development prospects	Career development	Difficulty in job promotion Wasting of technical skills
		Interpersonal relationships	Lack of leadership attention Complex relationships among colleagues
		Talent cultivation	Few opportunities for advanced training Average technical support from higher-level hospitals The absence of mentor-mentee models
Perceived Behavioral Control	External opportunities	Recruitment by higher-level medical institutions	Recruitment by higher-level medical institutions and moving to better hospitals after contract expiry
		Further studies or career change	Pursuing a master’s degree after the service period Planning to switch to other industries

### The attributions of turnover intentions influencing factors

#### Subjective norms

Subjective norms mean an individual’s perception of social expectations when he intends to engage in certain behavior. The subjective norms of rural-oriented medical students are primarily constituted by the expectations of their families and the perception of social cognition. Nanning, which adheres to its principle of “enrollment by designated regions”, recruits rural-oriented medical students from surrounding cities. However, the actual sources of students are not entirely balanced, which results in some rural-oriented students being assigned to township health clinics far from their homes, facing significant language differences across regions, and consequently, they are prone to experiencing a lower sense of belonging. Their families hope that they can return to work in their hometowns. Negative online opinions about the rural-oriented medical students project, along with the spread of pessimistic sentiments among the students themselves regarding their work in township health clinics, also impact the students’ perceptions about leaving these clinics after completing their service period.

#### Behavioural attitudes

Behavioural attitude refers to the disposition formed through the conceptualization of an individual’s evaluation of a specific behavior. Therefore, the components of attitude are often regarded as a function of the individual’s salient beliefs about the outcomes of this behavior. The behavioral attitudes of rural-oriented medical students are primarily constituted by Career Choice Deviation and Professional Realities. Rural-oriented medical students and their parents initially had an incomplete understanding of the policy. They focused only on benefits and failed to consider whether the policy truly suited students or their families. Plenty of Rural-oriented medical students felt a significant gap upon returning to township health center after completing their standardized training. They also felt that working in these clinics was unsuitable for them and the service period was too long. They are also unaware that rural-oriented medical students in the contract-based program are not allowed to pursue postgraduate studies during their service period. Disappointment with the professional reality also serves as a significant factor influencing the attitude of rural-oriented medical students towards turnover behavior. This included poor salaries and benefits in township health clinics, substandard accommodation conditions, frequent difficulties in doctor-patient communication, low levels of technical skills in work, and high workloads encompassing both clinical and public health responsibilities can lead to burnout among rural-oriented medical students. Rural-oriented medical students often feel neglected due to limited opportunities for external training during the three-year service period after standardized training, which is a consequence of staffing shortages in health clinics. Interpersonal relationships among colleagues also constitute another significant factor affecting the sense of belonging of rural-oriented medical students to their assigned township health clinics. Due to the high turnover of rural-oriented medical students in the past, both the leadership and colleagues at township health clinics tend to believe that these students will leave upon the completion of their service period. Although their educational level is higher than that of their surrounding colleagues, they are not given priority in professional development, leading to a low sense of affiliation among the rural-oriented medical students. Furthermore, challenges in job promotion and the lack of discipline and platform development in township health clinics, coupled with an overall low level of technical proficiency, led directed medical students to feel the waste of their skills and thereby generate resignation intentions. In addition, Inadequate drug and equipment resources compared to training hospitals also contribute to the rural-oriented medical students’ disappointment with the professional reality. All the aforementioned factors influence the attitude of rural-oriented medical students towards turnover behavior.

#### Perceived behavioural control

Perceived behavioral control indicates an individual’s perceived ease or difficulty in performing a specific behavior, which reflects their sense of control or mastery. External opportunities are identified as the primary factor influencing the difficulty level perceived by rural-oriented medical students in assessing their turnover behaviors. For students engaged in rural-oriented medical programs, pursuing opportunities such as recruitment by higher-level medical institutions, further studies, or a career change are perceived as relatively feasible options with minimal difficulty.

### The consequence of turnover intention

#### Siphoning by higher-level medical institutions

Being siphoned off by higher-level medical institutions constitutes a significant option for the turnover of students enrolled in rural-oriented medical programs. Due to the shortage of medical personnel in county-level and higher-tier hospitals, rural-oriented medical students seeking higher salaries and better working conditions are often siphoned off by these upper-level medical institutions.

#### Further studies or career change

Upon completion of their service period, many students from rural-oriented medical programs opt to pursue graduate studies or switch careers. Pursuing postgraduate studies enhances their prospects of securing positions in urban tertiary hospitals. Meanwhile, a portion of the rural-oriented medical students, having grown weary of medical practice, contemplate career shifts to pharmaceutical companies or opt out of the healthcare sector altogether.

## Discussion

This study develops a model based on the Theory of Planned Behaviour, delineating the contributing factors and their interactive mechanisms influencing the turnover intentions of rural-oriented medical students upon completion of their service period, Compared to previous investigations into the factors influencing turnover among China’s rural-oriented medical students, the model in this study is constructed on the basis of Grounded Theory rather than through questionnaire-based research, offering a more comprehensive exploration of the mechanisms behind the formation of turnover intentions among rural-oriented medical students. This model can serve as a reference for the subsequent formulation of policies in China, aimed at retaining a greater number of rural-oriented medical students at the grassroots level.

The formation of turnover intentions is primarily composed of three elements: Subjective Norms, Behavioral Attitudes, and Perceived Behavioral Control. Within this framework, Subjective Norms are equated to Public Opinion, Behavioral Attitudes comprise Career Choice Deviation, Professional Realities, and Development Prospects, and Perceived Behavioral Control is represented by External Opportunities. Currently, most studies on the turnover intentions of rural-oriented medical students emphasize Behavioral Attitudes, with a particular focus on Professional Realities. Amalba *et al*. identified the primary deterrents to choosing rural employment as the scarcity of recreational facilities, limited career development opportunities due to financial and material resources, and insufficient coverage of rural practice in the curriculum (Amalba *et al.*, 2020). Nallala *et al*. identified that good housing, better salaries, and adequate facilities are significant factors influencing medical students’ decisions to work in rural areas (Nallala *et al.*, 2015). Liu *et al*. conducted a questionnaire survey among 4,278 rural-oriented medical students in China and discovered a significant correlation between the internship period of these students and their intention to work in rural areas (Liu *et al.*, 2018). Zhang *et al*. identified that income, working hours, training opportunities, working conditions, and career advancement are the primary factors influencing the turnover intentions of rural-oriented medical students upon the completion of their service period (Zhang *et al.*, 2022). In general, township health center with weak operational autonomy has lower salary levels and less performance-related pay (Li *et al.*, 2021; Wang *et al.*, 2022). Several Chinese scholars have found that the operational level of township health centers also plays a significant role in influencing the turnover intentions of rural-oriented medical students (Chen *et al.*, 2022; Luo *et al.*, 2022). township health center with specialized departments and better economic performance can retain rural-oriented medical students after their service period. Research has also found that effectively enhancing organizational identification can reduce their desire to resign.

This model incorporates the elements of Subjective Norms and Perceived Behavioral Control. These components encompass the unique circumstances encountered in the actual implementation of the RTME program in China. Due to the incomplete establishment of China’s tiered healthcare system, there is a pronounced siphoning effect by urban tertiary hospitals, resulting in a shortage of human resources in county-level public hospitals and primary healthcare institutions. Some rural-oriented medical students are siphoned off to county-level hospitals or higher-tier medical institutions. Rural-oriented medical students are often siphoned off to county-level hospitals or higher-tier medical institutions, Currently, urban tertiary hospitals predominantly recruit candidates with master’s or doctoral degrees, prompting medical students to pursue further education at the master’s level to access higher-paying jobs with better development prospects in these top-tier urban hospitals. The current policy merely restricts the pursuit of postgraduate studies and employment in other medical institutions during the service period. However, realizing these two external opportunities is not challenging for rural-oriented medical students. Subjective norms significantly influence the genesis of turnover intentions among rural-oriented medical students. Rural-oriented medical students and their parents initially had an incomplete understanding of the policy. They focused only on benefits and failed to consider whether the policy truly suited students or their families. Plenty of rural-oriented medical students felt a significant gap upon returning to township health clinics after completing their standardized training. A study from Gansu Province showed that the willingness of rural-oriented medical students to work in rural areas was limited and mainly influenced by external rather than internal motivations, as most didn’t fully understand the policy. Therefore, it is recommended to offer more comprehensive policy promotion and interpretation to prospective students and their parents before the recruitment of directed medical students. some rural-oriented medical students are not working in the township health centers of their hometowns. The desire of their families for these students to return and work in their native places contributes to the emergence of their turnover intentions. However, the mobility mechanism for rural-oriented medical students between township health center is not fully established. It is recommended to facilitate the mobility of rural-oriented medical students between nearby township health clinics and adjust subsequent enrollment quotas to achieve a balanced total number across regions little by little.

## Limitations of the study

In this study, qualitative research methods were employed to construct turnover intentions, lacking a quantitative survey on the population sociology factors of rural order-oriented medical students’ turnover intentions. The next step could involve incorporating demographic and sociological factors into this model through a combination of qualitative and quantitative methods.

## Conclusion

This paper systematically developed a mechanistic model for turnover intentions among rural-oriented medical students using grounded theory, providing a fresh perspective. It further delves into how Subjective norms, Behavioral attitudes, and Perceived Behavioral Control affect the emergence of turnover intentions and explores their interplay. The results introduce Subjective norms and Perceived Behavioral Control as novel components to the model, thereby enhancing its comprehensiveness. As a result, this model can serve as a valuable reference for future policy optimization concerning China’s rural order-directed medical students.

## Data Availability

No additional data are available.
